# Effect of Preoperatively Continued Aspirin Use on Early and Mid-Term Outcomes in Off-Pump Coronary Bypass Surgery: A Propensity Score-Matched Study of 1418 Patients

**DOI:** 10.1371/journal.pone.0116311

**Published:** 2015-02-23

**Authors:** Fucheng Xiao, Hengchao Wu, Hansong Sun, Shiwei Pan, Jianping Xu, Yunhu Song

**Affiliations:** Department of Cardiovascular Surgery, State Key Laboratory of Cardiovascular Disease, Fu Wai Hospital and Cardiovascular Institute, National Center for Cardiovascular Diseases, Peking Union Medical College and Chinese Academy of Medical Sciences, Beijing, China; Thomas Jefferson University, UNITED STATES

## Abstract

**Background:**

To date, effect of preoperatively continued aspirin administration in off-pump coronary artery bypass grafting (CABG) is less known. We aimed to assess the effect of preoperatively continued aspirin use on early and mid-term outcomes in patients receiving off-pump CABG.

**Methods:**

From October 2009 to September 2013 at the Fuwai Hospital, 709 preoperative aspirin users were matched with unique 709 nonaspirin users using propensity score matching to obtain risk-adjusted outcome comparisons between the two groups. Early outcomes were in-hospital death, stroke, intra- and post-operative blood loss, reoperation for bleeding and blood product transfusion. Major adverse cardiac events (death, myocardial infarction or repeat revascularization), angina recurrence and cardiogenic readmission were considered as mid-term endpoints.

**Results:**

There were no significant differences among the groups in baseline characteristics after propensity score matching. The median intraoperative blood loss (600 ml versus 450 ml, P = 0.56), median postoperative blood loss (800 ml versus 790 ml, P = 0.60), blood transfusion requirements (25.1% versus 24.4%, P = 0.76) and composite outcome of in-hospital death, stroke and reoperation for bleeding (2.8% versus 1.6%, P = 0.10) were similar in aspirin and nonaspirin use group. At about 4 years follow-up, no significant difference was observed among the aspirin and nonaspirin use group in major adverse cardiac events free survival estimates (95.7% versus 91.5%, P = 0.23) and freedom from cardiogenic readmission (88.5% versus 85.3%, P = 0.77) whereas the angina recurrence free survival rates was 83.7% and 73.9% in the aspirin and nonaspirin use group respectively (P = 0.02), with odd ratio for preoperative aspirin estimated at 0.71 (95% confidence interval, 0.49-1.04, P = 0.08).

**Conclusions:**

Preoperatively continued aspirin use was not associated with increased risk of intra- and post-operative blood loss, blood transfusion requirements and composite outcome of in-hospital death, stroke and reoperation for bleeding in off-pump CABG. Preoperative aspirin use tended to decrease the hazard of mid-term angina recurrence.

## Introduction

Aspirin is a mainstay treatment for patients with coronary artery disease worldwide. The benefit of early-postoperative aspirin administration is clear given on preventing graft failure [1], reducing ischemic complication (myocardial infarction and stroke) and improving survival [2] in patients undergoing coronary artery bypass grafting (CABG). However, there are conflicting evidences whether or not the benefits of preoperative aspirin ingestion up to the time of surgery may outweigh the hazards of excessive bleeding and transfusion requirements. Based on favorable evidences from observational studies, including decreased operative mortality [3–4] and morbidity [5] and improved graft patency [6] of preoperatively continued aspirin use, the American College of Cardiology and American Heart Association latest 2011 guidelines [7] for CABG recommended that aspirin (100 mg to 325 mg daily) should be administered to CABG patients preoperatively. In contrast, given the increasingly competing risk of hemorrhage-related complications [8–10] (postoperative blood loss, reoperation for bleeding and transfusion requirement) associated with preoperatively continued aspirin use, the Society of Thoracic Surgeons latest 2012 guidelines [11] gave a class IIa recommendation to the discontinuation of aspirin before purely elective CABG in patients without acute coronary syndrome. Moreover, to date most of those studies have been done in patients undergoing conventional on-pump CABG. The effect of preoperative aspirin administration remains unclear in off-pump CABG. With an increasing volume of Off-pump CABG performed in Asian countries which accounts for at least 60% of all the CABG [12] and in consideration of the significant differences in term of postoperative coagulation system between off-pump CABG and on-pump CABG, it is essential to evaluate the preoperatively continued aspirin use until surgery in patients receiving off-pump CABG with regard to in-hospital mortality, stoke and hemorrhage-related complications. Additionally, the mid-term effect of preoperatively continued aspirin administration in patients undergoing off-pump CABG has not been investigated.

To address those issues, we performed a retrospective study to evaluate the association between preoperatively continued aspirin ingestion until surgery and postoperative complications and mid-term outcomes in patients receiving off-pump CABG.

## Materials and Methods

### Patient population

The present analysis is based on a consecutive series of patients who underwent isolated off-pump CABG from October 2009 to September 2013 at the Fuwai Hospital, Beijing, China. Preoperatively continued aspirin use (100mg/daily) is defined as aspirin ingestion up to the time of operation. Prior to October 2009, preoperative aspirin was routinely stopped for at least 5 days before elective off-pump CABG in our center. Thereafter, preoperative aspirin use was left to the discretion of individual surgeon. During the study period, 5787 patients with only aspirin use for anti-platelet therapy preoperatively underwent elective isolated off-pump CABG at our institution and the in-hospital mortality was 12 (0.2%). Of those 5787 patients, 720 patients continued aspirin use preoperatively. We propensity-matched (see statistical analysis) 709 out of 720 patients taking continued aspirin therapy to 709 out of 5067 patients whose aspirin was withdrawn for more than 5 days before operation. A cohort of 709 propensity-matched pairs of patients constituted the study population. Patients who were exposed preoperatively to warfarin or other antiplatelet agents such as clopidogrel or dipyridamole were excluded from this study, as well as those who underwent emergent operation. Approval for the study was obtained from Institutional Review Board of Fuwai Hospital with patient consent waived.

### Surgical procedure

All the off-pump CABGs were performed by experienced surgeons. As part of standard institutional requirements, all of them had to have specialized in congenital or valve heart surgery for at least 3 years before undertaking any CABG procedures. With respect to off-pump CABG, the surgeon had to perform at least 100 on-pump CABG procedures before being considered qualified to carry out the off-pump procedure. The heart was exposed through a median sternotomy in all the patients. An Octopus stabilizer (Medtronic, Minneapolis, MN), a humidified carbon dioxide blower (Medtronic DLP, Grand Rapids, MI) and intracoronary shunts (CardioThoracic System, Cupertino, CA, USA) were used routinely to facilitate the anastomoses distally or proximally. The proximal anastomoses were sutured to the partial-clamped ascending aorta when controlled hypotension was maintained at a low and stable level of 80–90 mmHg for systolic blood pressure and about 60 mmHg for mean blood pressure. All patients had mediastinal and pericardial drains or pleural drains if necessary. Generally, the drains were removed on the second postoperative day when drainage was less than 20 mL for 5 to 6 consecutive hours. Blood transfusion was indicated if the hemoglobin level was less than 90g/L. If the blood loss was more than 200 ml/hour for 3 to 4 consecutive hours, chest reoperation for bleeding was performed.

### Perioperative anticoagulant management

Unfractionated heparin (200 IU/kg body weight) was administered before harvesting the left internal mammary artery. Additional heparin was administered to maintain the activated clotting time>300 seconds throughout the operation. On completion of all anastomoses, heparin was routinely neutralized with protamine sulfate at a ratio of 0.6:1 (protamine to heparin). Further, protamine was administered for bleeding during closure of the chest or within the first hour after surgery according to the activated coagulation time. Tranexamic acid was administered intraoperatively according to the preference of the anesthesiologist. Preoperative nonaspirin users were put on prophylactic subcutaneous heparin at the time of hospitalization. Postoperative aspirin (100mg/daily) was generally administrated at 6 hours postoperatively.

### Clinical end points and definition

The early outcome measurements of our study were intra- and post-operative blood loss, blood product transfusion (both intraoperatively and postoperatively) and individual or composite outcome of in-hospital death, stroke and reoperation for bleeding. Major adverse cardiac events (MACE: death, myocardial infarction or repeat revascularization), angina recurrence and the need for rehospitalization due to cardiac reasons during follow-up were considered as mid-term endpoints. In-hospital death was defined as death during hospitalization regardless of cause. Standard definitions of myocardial infarction and stroke defined according to the Society of Thoracic Surgeons database [13] were used. Reoperation for bleeding was defined as bleeding requiring surgical intervention after surgery. Stroke was defined as a central neurologic deficit persisting postoperatively for > 72 hours. We excluded confused states, transient events, and intellectual impairment to avoid any subjective bias. Death during follow up was defined as all-cause death occurred after patient’s discharge. Myocardial infarction during follow-up was defined a new Q wave in two or more contiguous leads on electrocardiography, or significant increase of cardiac enzyme levels (great than the upper limit of the normal) combined with electrocardiographic or clinical or angiographic evidence of myocardial infarction. Repeat revascularization was defined as percutaneous coronary intervention or coronary artery bypass grafting. Recurrence of angina was defined as occurrence of chest pain or distress due to myocardial ischemia after discharge. Cardiogenic rehospitalization was defined as readmission due to cardiac reasons.

### Clinical follow-up

The postoperative results were assessed in all patients at discharge and during follow-up. All patients had been followed up at least 3 months since discharge from hospital with the exception of patients who were lost to follow-up. Follow-up involved review of outpatient and (or) inpatient medical records and structured telephone interviews. The medical records in outpatient clinics of those who reported any adverse events after discharge were reviewed for further confirmation. When any major adverse event was reported by another hospital, patients were requested to mail a copy of all relevant medical information. Patients diagnosed with hyperlipemia resumed statins therapy and beta-blocker was generally taken in all the patients except for few patients with bradycardia during follow-up. Follow-up was 97.7% (693/709) complete in aspirin users versus 96.3% (683/709) in nonaspirin users (p = 0.12), with a mean follow-up of 22.8, 22.0 and 22.6 months for major adverse cardiac events, angina recurrence and readmission due to cardiac events respectively. The data of the patents who were lost to follow up was only included in the in-hospital outcome analysis.

### Statistical analysis

To minimize potential selection bias in the comparisons of outcomes between the two groups, propensity score matching using multivariate logistic regression model was performed to match each preoperative aspirin user with unique nonaspirin user in a 1-to-1 fashion. To maximize the pair-matching among the two groups, variables selected for inclusion in the propensity score were core patient characteristics, which were unevenly distributed among two groups before propensity matching, including age, sex, insulin-dependent diabetes, hypertension, and number of vessels grafted. The way in which we included variable in the propensity score matching was described previously [5]. The goodness-of-fit of this logistic regression model was appraised using Hosmer-Lemeshow test (p = 0.88). Using a greedy matching algorithm [14], patients with preoperative aspirin use were matched with nonaspirin users on an identical propensity score in a higher-digit priority order, namely a five-digit to a four-, three-, two-, or one-digit match. If more than one non-aspirin patient were matched to an aspirin user patient, nonaspirin user was selected randomly among those patients and eventually, 709 well-matched pairs of patients were obtained. Thereafter, early and mid-term outcomes were compared to evaluate group differences.

Continuous data are shown as mean ± standard deviation. The student t test was used to measure the differences for variables with a normal distribution and equal variances. The Wilcoxon rank sum test was used for variables not normally distributed. Categorical data are displayed as frequencies and percentages and comparisons were made with Chi-square tests (Fisher exact tests if appropriate). P values <0.05 was considered statistically significant. Kaplan-Meier product limit curves for event-free survival were constructed and compared with the log-rank test. The odd ratio (OR) with 95% confidence interval (CI) was derived from the Cox proportional hazards model. All statistical analyses were performed using SAS for Windows version 9.1 (SAS Institute, Cary, NC).

## Results

### Group demographics and procedural characteristics

The demographic, clinical and procedural data of aspirin and nonaspirin users before and after propensity score matching are shown in Table 1. After propensity score matching, both groups were well matched, with the only significant difference being that patients on preoperative aspirin therapy had more patients with history of angina (p = 0.04). Preoperative aspirin users were associated with a trend toward having more patients of left main trunk disease, but such difference did not reach statistical significance. There was no significant difference between the two groups with regard to age, number of women, body mass index, insulin-dependent diabetes, hypertension, renal dysfunction, hyperlipidaemia, chronic obstructive pulmonary diseases and etc. The mean number of distal anastomoses were 3.3±0.8 in the patients group who discontinued aspirin for more than 5 days before surgery versus 3.2±0.8 in the continued aspirin therapy group (p = 0.37). The preoperative hemoglobin concentration was 135.1±15.2 g/L and 136.1±16.0 g/L in the nonaspirin and aspirin users group respectively (p = 0.11). The two groups were similar in the use of left internal mammary artery (p = 0.67), and number of prior cardiac surgery (p = 0.28). The ratio of patients with preoperative aspirin use to those without was 0.82 (85/104), 0.85(140/165), 1.10 (210/191) and 1.10 (274/249) during the period of 2009.10–2010.9, 2010.10–2011.9, 2011.10–2012.9 and 2012.10–2013.9 respectively (p = 0.11).

**Table 1:** Demographics and procedural characteristics of patients based on preoperative aspirin use.

	Overall patient			Pairs matched by PS		
	Nonaspirin users (n = 5067)	Aspirin users (n = 720)	P	Nonaspirin users (n = 709)	Aspirin users (n = 709)	P
Age, (y)*	59.7±8.1	61.5±9.0	<0.01	61.7±8.9	61.6±8.9	0.97
Female*	886(17.5%)	160(22.2%)	<0.01	164(23.1%)	157(22.1%)	0.65
**BMI, (kg/m** ^**2**^)	26.0±2.9	25.5±3.0	0.18	25.7±3.2	25.6±3.1	0.84
**History of smoking**	2483(49.0%)	370(51.4%)	0.23	341(48.1%)	368(51.9%)	0.15
Diabetes mellitus*	1647(32.5%)	258(35.8%)	0.08	252(35.5%)	258(36.4%)	0.74
Hypertension*	3192(63.0%)	490(68.1%)	<0.01	483(68.1%)	481(67.8%)	0.90
**Renal dysfunction**	56(1.1%)	9(1.3%)	0.73	5(0.7%)	9(1.3%)	0.28
**COPD**	162(3.2%)	21(2.9%)	0.69	21(3.0%)	19(2.7%)	0.75
**Carotid artery stenosis**	927(18.3%)	142(19.8%)	0.32	135(19.0%)	138(19.6%)	0.81
**Cerebrovascular disease**	578(11.4%)	91(12.6%)	0.33	87(12.3%)	91(12.8%)	0.75
**Hyperlipidaemia**	3047(60.1%)	420(58.3%)	0.36	411(58.0%)	416(58.3%)	0.50
**Angina**	4611(91.0%)	675(93.8%)	0.02	649(91.5%)	669(94.4%)	0.04
**Myocardial infarction**	2037(40.2%)	286(39.7%)	0.80	298(42.0%)	280(39.5%)	0.33
**Ejection fraction, (%)**	60.1±8.6	59.6±9.2	0.57	60.5±8.4	59.7±9.5	0.38
**LVEDD, (mm)**	48.5±5.9	49.4±5.9	0.58	49.5±5.9	49.5±5.8	0.96
**RWMA**	1829(36.1%)	281(39.4%)	0.07	250(35.3%)	276(39.3%)	0.12
**Atrial fibrillation**	137(2.7%)	17(2.4%)	0.61	21(3.0%)	16(2.3%)	0.42
**Ventricular arrhythmia**	127(2.5%)	14(2.0%)	0.37	15(2.1%)	14(2.0%)	0.86
**Pacemaker implantation**	36(0.7%)	2(0.3%)	0.22	4(0.6%)	2(0.3%)	0.41
**Prior cardiac surgery**	43(0.9%)	10(1.4%)	0.15	5(0.7%)	9(1.3%)	0.28
**Coronary artery disease**			0.12			0.06
**Single-vessel disease**	65(1.3%)	14(2.0%)		11(1.6%)	13(1.9%)	
**Two-vessel disease**	468(9.3%)	51(7.1%)		75(10.6%)	51(7.2%)	
**Three-vessel disease**	2784(55.1%)	391(54.7%)		398(56.3%)	384(54.6%)	
**Left main truck disease**	1736(34.4%)	259(36.2%)		223(31.5%)	256(36.4%)	
**Preoperative Hb, (g/L)**	136.3±15.0	136.5±16.1	0.26	135.1±15.2	136.1±16.0	0.11
Distal anastomoses*	3.6±1.0	3.2±0.9	<0.01	3.3±0.8	3.2±0.8	0.37
**LIMA**	4813(95.0%)	694(96.4%)	0.10	681(96.1%)	684(96.3%)	0.67
**NYHA**			0.11			0.19
**1**	462(9.2%)	80(11.2%)		56(7.9%)	80(11.3%)	
**2**	4122(81.8%)	559(78.2%)		575(81.3%)	555(78.5%)	
**3**	421(8.4%)	68(9.5%)		70(9.9%)	67 (9.5%)	
**4**	35(0.7%)	8(1.1%)		6(0.9%)	5(0.7%)	

BMI indicates body mass index; COPD, chronic obstructive pulmonary disease; Hb, hemoglobin; LIMA, left internal mammary artery; LVEDD, left ventricular end diastolic diameter; NYHA, New York Heart Association; PS, propensity score; RWMA, regional wall motion abnormity; SD, standard deviation

*Variable included in the regression model for propensity score matching.

### In-hospital outcomes

Results of in-hospital outcomes are given in Table 2. No significant differences was observed in early composite endpoints (in-hospital death, stroke and reoperation for bleeding), with 11 total events (1.6%) in preoperative nonaspirin users versus 20 (2.8%) in preoperative aspirin users (p = 0.10). Also, there were no significant differences between preoperative nonaspirin and aspirin therapy group with regard to in-hospital mortality (0.1% versus 0.1%, p = 1.00), stroke (0.1% versus 0.3%, p = 1.00), intraoperative blood loss (450 ml versus 600 ml, p = 0.56), postoperative blood loss (790 ml versus 800 ml, p = 0.60), and duration of intubation (17.6±10.2 h versus 17.6±11.7 h, p = 0.15). Although not statistically significant, the rate for reoperation for bleeding was higher in aspirin users group (1.3% versus 2.4%, p = 0.11). In patients with blood transfusion, the median intraoperative blood loss with 25th and 75th centiles was 300 ml (300－610 ml) and 600 ml (300－750 ml) in nonaspirin and aspirin users group respectively (p = 0.30) whereas the median postoperative blood loss was 1020 ml (340－2940 ml) and 1130 ml (290－3700 ml) respectively (p = 0.17). The results of blood product requirements are summarized in Table 3. There were no significant differences among the two groups in blood transfusion rate (24.4% versus 25.1%, p = 0.76) and transfusion requirements of red blood cells, platelets and fresh frozen plasma.

**Table 2:** In-hospital outcomes of propensity-matched patients based on preoperative aspirin use.

	Nonaspirin users(709)	Aspirin users(709)	P
**Composite in-hospital outcome, n (%)**	11(1.6%)	20(2.8%)	0.10
**In-hospital mortality, n (%)**	1(0.1%)	1(0.1%)	1.0
**Stroke, n (%)**	1(0.1%)	2(0.3%)	1.0
**Reoperation for bleeding, n (%)**	9(1.3%)	17(2.4%)	0.11
**Duration of intubation,(h), (mean±SD)**	17.6±10.2	17.6±11.7	0.15
**Intraoperative blood loss, (ml)**	450(200–1000)	600(260–1000)	0.56
**Postoperative blood loss, (ml)**	790 (200–2940)	800(210–3700)	0.60

SD indicates standard deviation; composite in-hospital outcome includes in-hospital mortality, stroke and reoperation for bleeding. Blood loss is shown as median with 25th and 75th centiles.

**Table 3:** Blood product requirements of propensity-matched patients based on preoperative aspirin use.

Types of blood products	Nonaspirin users(709)	Aspirin users(709)	P
**Red blood cells (units)**			0.62
**0**	576(81.2%)	567(80.0%)	
**2**	86(12.1%)	81(11.4%)	
**3**	0(0%)	1(0.1%)	
**4**	27(3.8%)	35(4.9%)	
**≥5**	20(2.8%)	25(3.5%)	
**Fresh frozen plasma (ml)**			0.18
**0**	623(88.0%)	609(86.0%)	
**200**	9(1.3%)	8(1.1%)	
**400**	45(6.4%)	66(9.3%)	
**600**	9(1.3%)	5(0.7%)	
**800**	7(1.0%)	11(1.6%)	
**≥1000**	16(2.3%)	10(1.4%)	
**Platelets (units)**			0.14
**0**	705(99.4%)	699(98.6%)	
**1**	3(0.4%)	9(1.3%)	
**≥2**	1(0.1%)	1(0.1%)	
**Transfusion, n (%)**	173(24.4%)	178(25.1%)	0.76

### Mid-term outcomes

**Fig 1:**
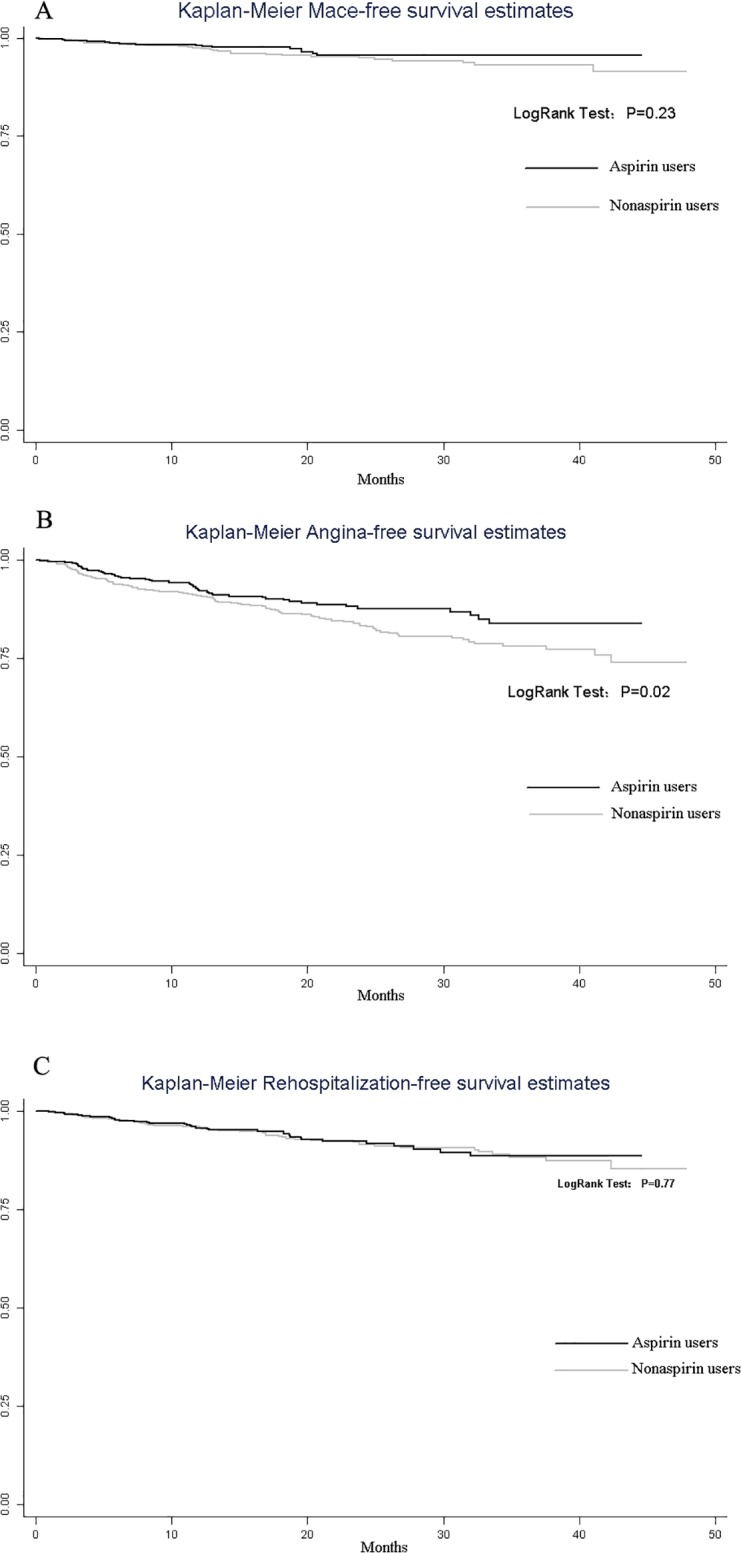
Event-Free Kaplan-Meier Estimates for Preoperative Aspirin and Nonaspirin Use Group. Shown are percent survival free from Mace (A), survival free of Angina recurrence (B) and survival free from rehospitalization due to cardiac reasons(C). MACE: major adverse cardiac event.

**Fig 2:**
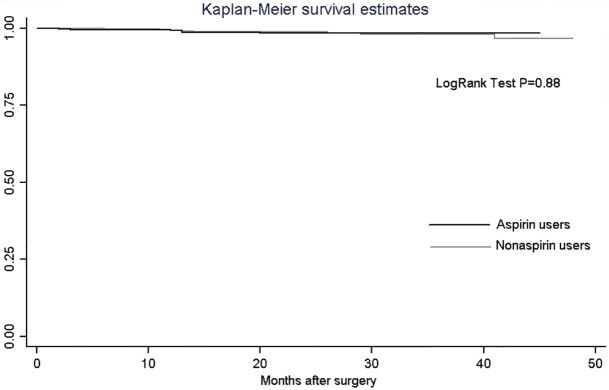
Kaplan-Meier Survival Estimates for Preoperative Aspirin and Nonaspirin Use Group.

There were 88 (12.4%) and 73 (10.3%) patients under dual antiplatelet treatment with aspirin and clopidogrel during follow-up in the aspirin and nonaspirin use groups respectively (p = 0.21). Details of mid-term outcomes during follow-up are described in Table 4. Major adverse cardiac events (MACE: death or myocardial infarction or repeat revascularization, percutaneous in all cases) occurred in 18 (2.5%) aspirin users and 30 (4.2%) nonaspirin users (p = 0.08). The Kaplan-Meier Mace-free survival estimates at about 4 years were 95.7% and 91.5% in the aspirin use group and nonaspirin use group respectively (p = 0.23; Fig. 1A). The OR for preoperative aspirin was estimated at 0.84 (95% CI, 0.41–1.71, p = 0.63). The survival rates free of angina recurrence at about 4 years was 83.7% in the aspirin use group versus 73.9% in the nonaspirin use group (p = 0.02; Fig. 1B), with OR for preoperative aspirin 0.71 (95% CI, 0.49–1.04, p = 0.08). No significant difference was found between aspirin and nonaspirin use groups with respect to the survival estimates free of readmission for cardiac reasons at about 4 years (88.5% versus 85.3% respectively, p = 0.77, Fig. 1C), with OR for preoperative aspirin 0.95 (95% CI, 0.57–1.56, p = 0.83). Maximum survival defined as the period between the first postoperative day until the last available follow-up date was given in Fig. 2.

**Table 4:** Mid-term outcomes of propensity-matched patients based on preoperative aspirin use.

	Nonaspirin users(709)	Aspirin users(709)	P
**Follow-up rate**	96.3%	97.7%	0.12
**MACE, n (%)**	30 (4.2%)	18 (2.5%)	0.08
**Death, n (%)**	9 (1.3%)	8 (1.1%)	0.81
**Repeat revascularization, n (%)**	12 (1.7%)	5 (0.7%)	0.09
**Myocardial infarction, n (%)**	11 (1.6%)	5 (0.7%)	0.13
**Angina recurrence, n (%)**	102 (14.4%)	61 (8.6%)	<0.01
**Cardiogenic readmission, n (%)**	50 (7.1%)	39 (5.5%)	0.23

MACE indicates major adverse cardiac events, including death, repeat revascularization and myocardial infarction.

## Discussion

The key finding of the present study is that preoperatively continued aspirin use was not associated with increased risk of intro- and post-operative blood loss, blood transfusion requirements, individual or composite outcomes of in-hospital mortality, stroke and reoperation for bleeding in patients who had off-pump CABG. Furthermore, our results suggest that preoperative aspirin administration was associated with improved angina-free survival and tended to decrease the mid-term hazard of angina recurrence.

The issue of preoperative aspirin therapy has been debated extensively in the literatures. To date, nearly all those studies regarding the effect of preoperative aspirin administration have been conducted in patients undergoing conventional on-pump CABG and have yielded inconsistent results. Arguments to withdraw preoperative aspirin therapy include the potential for increased bleeding and transfusion requirements postoperatively [8–10]. Several studies, however, suggest that continuing use of aspirin preoperatively reduce operative morbidity and mortality without increasing bleeding-related complications and transfusion requirement [3–5, 15]. The pooled results of recent meta-analysis [16–17] have reported the increased risk of bleeding and the need for blood transfusion in patients with late or no discontinuation of aspirin prior to CABG. However, these findings need to be treated with caution inasmuch as these analyses included studies that were performed over 30 years, with great variation in operative and anesthesiology methods and pharmacologic treatments. Additionally, the varying quality of the studies, the various aspirin dosages and the different time of discontinuation further complicated the interpretation of the pooled results. It is worth noting that in recent studies, preoperative aspirin therapy is even associated with a significant decrease in the risk of major cardiocerebral complications and 30-day mortality in patients undergoing cardiac surgery [18–19], particularly its’ renal protective effect in patients with chronic kidney disease undergoing cardiac surgery [20].

It is quite possible that preoperative aspirin use could increase the hazard of bleeding-related complications and the need for blood transfusion requirements in patients receiving conventional on-pump CABG due to clotting disorder, platelet dysfunction or systemic inflammatory response induced by cardiopulmonary bypass [21–23]. This, to some extent, may make preoperative aspirin therapy redundant for conventional on-pump CABG. The present study is carried out in patients undergoing off-pump CABG which obviates the need of cardiopulmonary bypass. Several reports documented that procoagulant or event hypercoagulable state would be developed after off-pump CABG probably attributed to much better preserved hemostasis in off-pump CABG compared with conventional on-pump CABG [21, 24–25]. The systematic review of 50279 patients taking aspirin for secondary prevention reported by Biondi-Zoccai et al [26] indicated that aspirin withdrawal was associated with three-fold higher risk of major adverse cardiac events (OR = 3.14, 95% CI, 1.75–5.61, p = 0.0001). It is speculated that, by virtue of platelet inhibition and antiinflammatory action of aspirin, keeping patients on continuing aspirin therapy prior to off-pump CABG may help attenuate the procoagulant or hypercoagulable state during the operative period and may also reduce thrombotic events while awaiting surgery. The issue whether the benefits of preoperative aspirin use may exceed the risk in patients undergoing off-pump CABG is of great importance and of much concern. In the first place, the effect of preoperative aspirin use on off-pump CABG has seldom been detailed. In addition, it is a frequently encountered clinical question which needs to be answered urgently due to an increasing volume of Off-pump CABG performed in Asian countries which accounts for at least 60% of all the CABG [12].

In the present study of 1418 patients undergoing off-pump CABG, we found there was no significant difference between the two groups in in-hospital mortality, stroke, intra- and post-operative blood loss, blood transfusion requirements and duration of intubation. Although not statistically significant, the rate for reoperation for bleeding was doubled in aspirin users group. With regard to mid-term endpoints during follow-up, no significant difference was observed among those two groups in Mace-free survival estimates and survival estimates free of rehospitalization for cardiac reasons. The present study also shows that despite of significant angina-free survival benefit associated with preoperative aspirin use in Kaplan-Meier survival analysis, but such difference did not reach significance in Cox proportional hazards regression analysis, with only a trend of preoperative aspirin use to decrease the mid-term hazard of angina recurrence. Additionally, although off-pump procedures were performed by several surgeons, it was not identified as a statistical significant factor in Cox analysis to affect those mid-term outcomes, with OR = 1.1 (95% CI:0.8–1.4; p = 0.46) for MACE occurrence, OR = 1.0 (95% CI:0.9–1.2; p = 0.69) for angina recurrence and OR = 1.1 (95% CI:0.8–1.3; p = 0.66) for cardiogenic readmission. Our results was in consistent with the results reported by Srinivasan et al [27], which was thus far the only report to appraise the effect of preoperative aspirin use on in-hospital outcomes in patients receiving off-pump CABG.

Due to the inconsistent recommendations from the aforementioned guidelines, the decision of preoperative aspirin use was varied among surgeons in our center. Surgeons were grouped into those who favored or disfavored or had no special requirement for preoperative aspirin use respectively. The surgeon’s decision on preoperative aspirin use was generally for all his patients, rather than for subjectively selected patients. However, under some clinical scenarios, surgeons who advocated discontinuation (continuation) of preoperative aspirin use would also perform off-pump CABG for patients with continuation (discontinuation) of preoperative aspirin use. Generally, those two groups were operated by the same group of surgeons. The reported in-hospital mortality from randomized controlled trials (RCTs) after off-pump CABG ranged from 1.5% up to 2.5% [28–29]. However, the in-hospital death of the present study is lower. We believed that this discrepancy could be primarily explained by reasons described below. There was difference in the definition of in-hospital mortality, which defined as all-cause mortality with 30-days after surgery or before discharge in the RCTs versus ours as death during hospitalization regardless of cause. The mortality rate of the former definition (operative mortality) in our cohort was 0.21% (3/1418), due to another death occurred after discharge (20 days after surgery) in aspirin users group. Low mortality similar to that of our study has also been reported. The annual report of coronary artery surgery from Japan shown that among 4936 patients receiving off-pump CABG in 2005, including high operative risk patients, the overall operative mortality was 0.6% [30]. Moreover, it seemed that our patient population was different from that of the RCTs, evidenced by enrolling more patients in the RCTs, with advanced age, diabetes, hypertension, renal dysfunction and etc, especially patients with urgent operation (16% and 40% respectively) who were excluded from our study. The mean EuroSCORE of our cohort and its predictive mortality was 1.8 and 1.1% respectively [31], indicating that on the whole, our study population was comprised of patients with low operative risk. Additionally, all the patients in our study were operated by experienced surgeons as described in surgical procedure section and the in-hospital mortality of the whole off-pump CABG patients between October 2009 and September 2013 in our hospital was 0.2% (12/5787)

It may appear strange that preoperative aspirin use affects the mid-term angina recurrence without much effect on MACE-free survival rate and rehospitalization-free survival estimates. This finding could be explained in part by the fact that aspirin has primarily been shown to prevent early graft failure caused mainly by thrombotic mechanisms induced by endothelial injury, leading to platelet and coagulation activation and thrombus formation. Thus, compared with preoperative aspirin user group, there is probably more early closure or stenosis of venous grafts in nonaspirin user group, resulting in more angina recurrence and eventually leading to survival curve free of angina recurrence diverging sharply during follow-up. Obviously, this is just a speculation and would require angiographic data to be confirmed. Also, it may well be that the period of follow-up of the present study is not long enough to detect any significant group difference of those severe outcomes of major adverse cardiac events and readmission for cardiac reasons in patients after CABG.

The present study has several strengths. The patient group in this study represents the largest group of patients studied with regard to the effect of preoperatively continued aspirin ingestion on hemorrhage-related outcomes and postoperative complications in patients undergoing off-pump CABG. Furthermore, our study was the first, to our knowledge, to assess some long-term benefits of continued aspirin use until surgery. However, there are also limitations in the present study. One major limitation was that it was a retrospective study from a single center, although propensity matching method, which is by far the best method of comparison in observational settings, was used, other unknown variables that affect the outcomes of interest could not be fully eliminated. Also, we were unable to adjust for antifibrinolytic use as the dosage of antifibrinolytic agents could not be obtained retrospectively due to incomplete data. Additionally, we did not have angiographic data to verify whether the improved survival curves free of angina recurrence was correlated with higher graft patency rate due to preoperatively continued aspirin use. Finally, the study might be underpowered. Although the study population was relatively large, the number of events for the early composite outcome was small, leading to a relatively insufficient sample size. Ideally, a large multi-center, randomized, controlled trial would be optimal to control for the unforeseen confounders and yield high-level evidences to verify our findings and to guide the clinical decision-making of preoperatively continued aspirin use.

In conclusion, preoperatively continued aspirin use was not associated with increased risk of intra- and post-operative blood loss, blood transfusion requirements and individual or composite outcome of in-hospital mortality, stroke and reoperation for bleeding in patients who had off-pump CABG. In addition, there was suggestive evidence from our study that preoperative aspirin administrated continually tended to decrease the mid-term hazard of angina recurrence. Thus, on the basis of the present study, we recommend that preoperative aspirin therapy should be ingested up until off-pump CABG without interruption.
